# Schisandrin B alleviates metabolic associated fatty liver disease by regulating the PPARγ signaling pathway and gut microbiota in mice

**DOI:** 10.3389/fphar.2025.1583307

**Published:** 2025-07-25

**Authors:** Jiuchen Wan, Chenjian Lang, Meng Gao, Feilong Liu, Xiyuan Feng, He Li, Chunmei Wang, Jinghui Sun

**Affiliations:** School of Pharmacy, Beihua University, Jilin, China

**Keywords:** Schisandrin B (Sch B), metabolic associated fatty liver disease (MAFLD), gut microbiota, gut barrier, PPARγ

## Abstract

**Objective:**

The aim of this study was to investigate the improving effect of Schisandrin B (Sch B) on metabolic associated fatty liver disease (MAFLD) by regulating the PPARγ signaling pathway and gut microbiota, and its mechanism in mice.

**Methods:**

Male C57BL/6 mice were fed with a high-fat diet (HFD) continuously for 16 weeks to establish a MAFLD model. The levels of aspartate aminotransferase (AST), alanine aminotransferase (ALT), triglycerides (TG), total cholesterol (TC), low-density lipoprotein cholesterol (LDL-C), high-density lipoprotein cholesterol (HDL-C), tumor necrosis factor-α (TNF-α), interleukin-6 (IL-6), interleukin-10 (IL-10), and lipopolysaccharide (LPS) in serum, as well as the level of malondialdehyde (MDA), and the activities of glutathione peroxidase (GSH-Px) and superoxide dismutase (SOD) in the liver tissue were measured. Changes in the gut microbiota of mice was analyzed by 16S rRNA sequencing technology. The expression levels of PPARγ, Plin2, Pck1, Acsl4, and Fads1 proteins, as well as those of zonula occludins 1 (ZO-1) and Occludin proteins in the colon tissue were detected by Western Blot.

**Results:**

The results showed that Sch B could alleviate the structure disorder, ballooning degeneration, inflammatory cell infiltration, liver lipid droplets, and fibrosis in liver tissue, lower the levels of AST, ALT, TG, TC, LDL-C, and LPS, increase the level of HDL-C and lower the levels of TNF-α and IL-6 in serum, increase the level of IL-10, and lower the level of MDA and increase the activities of SOD and GSH-Px in liver tissue in MAFLD mice. Sch B could increase the expression levels of PPARγ, Pck1, and Fads1 proteins, but decrease Plin2 and Acsl4 proteins in liver tissue. Sch B could improve the diversity and abundance of the gut microbiota, restore the normal composition of the gut microbiota at the phylum and genus levels, alleviate the disruption of the gut barrier caused by HFD, and enhance the expression of ZO-1 and Occludin proteins in colon tissue in MAFLD mice.

**Conclusion:**

This study showed Sch B can improve HFD-induced MAFLD, and the mechanism may be through regulating the PPARγ, Plin2, PCk1, Acsl4 and Fads1 signaling pathway, restoring the diversity of gut microbiota, and improving the gut barrier to delay the progression of MAFLD.

## Introduction

Metabolic associated fatty liver disease (MAFLD), formerly known as non-alcoholic fatty liver disease (NAFLD), refers to a type of chronic liver disease in which the hepatic cell steatosis and fat accumulation in the liver are induced by some metabolic disorders (Pipitone et al., 2023). MAFLD has become the most common chronic liver disease worldwide nowadays, and it can not only further transform into steatohepatitis, cirrhosis and even hepatocellular carcinoma, but also increase the mortality rate of liver-related diseases (Eslam et al., 2020). *Schisandra chinensis* (Turcz.) Baill. [*Magnoliaceae*] is the source of *Schisandrae (chinensis)* fructus - the mature and dried fruits and previousl weexlored whether preparations from the fruit can alleviate NAFLD was explored in our previous studies. The research results confirmed that lignans in *Schisandra chinensis* could alleviate glucose and lipid metabolism disorders, liver inflammation and lipid peroxidation, mitigate the liver lipid deposition, delay the progression of NAFLD and regulate the LXRα/SREBP-1c/FAS/ACC and SREBP2/HMGCR signaling pathways to affect the metabolism and transport of cholesterol and fatty acid in NAFLD animal models, and it was also speculated that peroxisome proliferators-activated receptors (PPARs) might be a potential target for *Schisandra chinensis* lignans to alleviate NAFLD (Liu et al., 2016; Sun et al., 2017). Schisandrin B (Sch B) was screened by molecular docking technology as a key component in *Schisandra chinensis* to alleviate MAFLD by regulating PPARγ in our previous works (Sun et al., 2022).

On the other hand, in recent years, the important impact of gut microbiota on human health has been widely recognized, and it has been found to directly or indirectly participate in many physiological processes (Wu et al., 2023), which makes gut microbiota a highly anticipated field in the research and potential treatment of MAFLD. Current research generally suggests that the specific impact of gut microbiota on MAFLD may involve impaired intestinal barrier function, LPS-induced inflammation and immune dysregulation, translocation of gut bacteria and their harmful metabolites, and gut microbiota-mediated bile acid dysregulation (Lian et al., 2020; Ji et al., 2020).

Therefore, this study was aimed to investigate the effects of Sch B on MAFLD mice and further analyze whether it could exert its effects via the PPARγ signaling pathway and by regulating gut microbiota, which was also expected to provide a basic data support for the subsequent development and utilization of *Schisandra chinensis*.

## Materials and methods

### Chemicals and reagents

Sch B (analytical reagent, purity >99.00%) was purchased from Chengdu Pufei De Biotech Co., Ltd. (Chengdu, China). Pioglitazone hydrochloride tablets were purchased from Chengdu Dikang Pharmaceutical Co., Ltd. (Chengdu, China). The basic feed and high-fat feed for mice were purchased from Changchun Yisi Experimental Animal Technology Co., Ltd. (Changchun, China), in which the components of the high-fat feed were as follows: lard (15%), sucrose (20%), cholesterol (1.2%), sodium cholate (0.2%), casein (10%), calcium hydrogen phosphate (0.6%), and basic feed (53%) (Feng et al., 2021). Alanine aminotransferase (ALT), aspartate aminotransferase (AST), total triglycerides (TG), total cholesterol TC, low-density lipoprotein cholesterol (LDL-C), high-density lipoprotein cholesterol (HDL-C), malondialdehyde (MDA), glutathione peroxidase (GSH-Px), and superoxide dismutase (SOD) biochemical kits were purchased from Nanjing Jiancheng Bioengineering Institute (Nanjing, China). Protein extraction kit, interleukin-6 (IL-6), interleukin-10 (IL-10), lipopolysaccharide (LPS), and tumor necrosis factor alpha (TNF-α) assay kits were purchased from Shanghai Enzyme Linked Biotechnology Co., Ltd. (Shanghai, China). HRP-Goat Anti-Rabbit IgG (H + L), PPAR-γ, Pck1, Fads1, Acsl4, Plin2, Occludin and GAPDH antibodies were all purchased from ABclonal Technology Co., Ltd. (Wuhan, China). ZO-1 antibody was purchased from Proteintech Group, Inc. (Wuhan, China). Chemiluminescence reagent was purchased from Meilunbio^®^ (Dalian, China).

### Animal grouping and administration

C57BL/6 male mice, weighing 18–22 g, were purchased from Changchun Yisi Experimental Animal Technology Co., Ltd. (Changchun, China), with the license number of SCXK (Ji) 2023-0002. Mice were kept in a sterile feeding room with a temperature controlled at 18°C–23°C and a humidity maintained at 40%–60%, and in an alternating light cycle for 12 h. The animal experiment was approved by the Experimental Animal Ethics Committee of Beihua University (Jilin, China; No. BULAEC-2023121202), and all experimental procedures were strictly carried out in accordance with the “Guide for the Care and Use of Laboratory Animals”.

**FIGURE 1:**
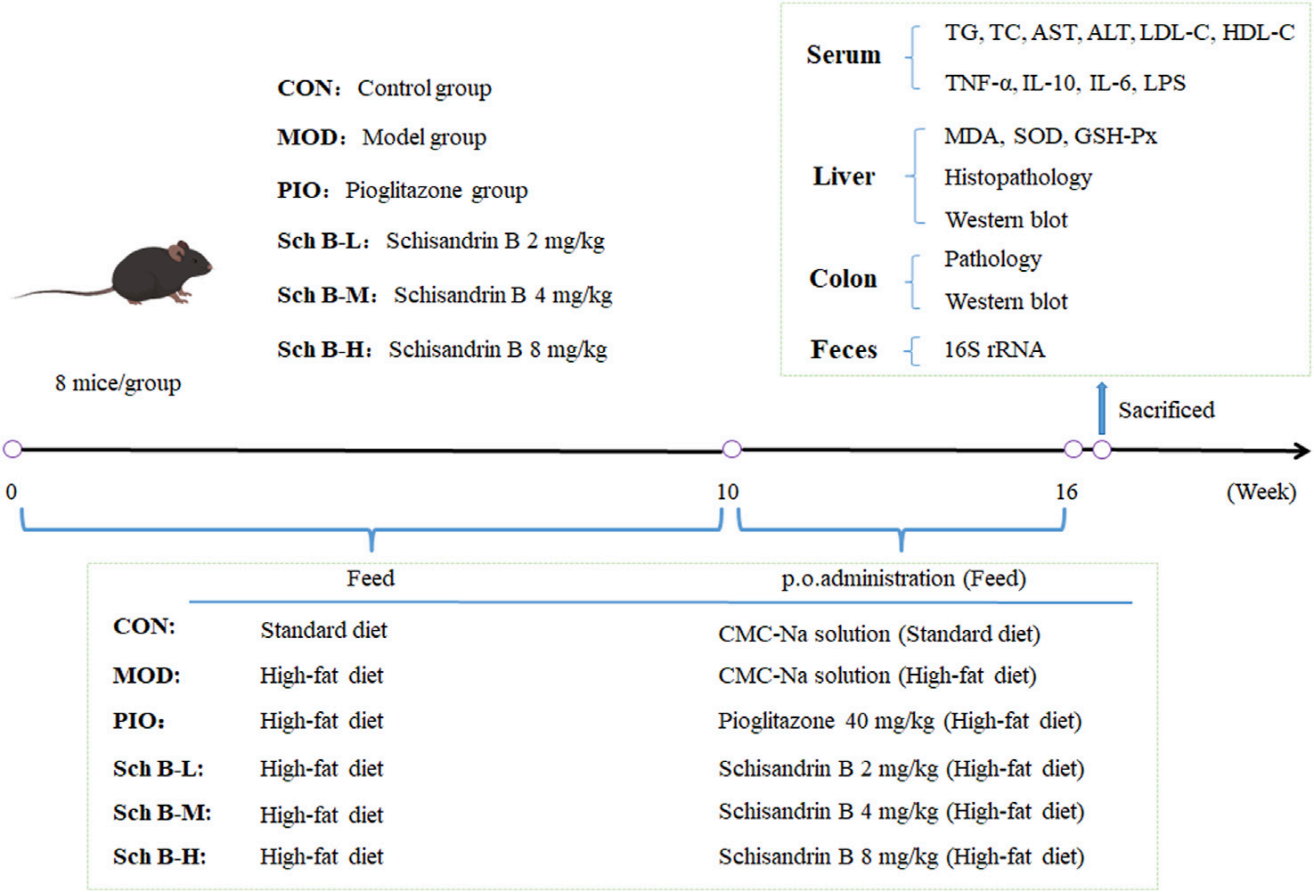
Grouping and administration of mice and experimental process.

After the adaptive feeding for 1 week, 48 mice were randomly divided into 6 groups: control group (CON), model group (MOD), pioglitazone group (40 mg/kg, PIO), Sch B-low dose (Sch B-L, 2 mg/kg) group, Sch B-middle dose (Sch B-M, 4 mg/kg) group, Sch B-high dose (Sch B-H, 8 mg/kg) group, 8 mice in each group. Mice in CON group were given the standard feed, and those in all other groups were fed with a high-fat diet (HFD) for 16 consecutive weeks. During the entire experiment, mice in all groups were free to eat feed and drink water. Mice in the CON group and MOD group were intragastrically given corresponding volumes of 0.5% carboxymethyl cellulose sodium (CMC-Na) solution, and those in the other groups were administered intragastrically with the corresponding doses of agents described as above from the 10th week. The mice were administered once a day continuously for 6 weeks. The weight of mice was recorded once weekly. After the last administration on the 16th week, fecal samples of mice were collected through the anus of mice using sterile EP tubes, and then stored in liquid nitrogen immediately. After fasting for 12 h, mice were anesthetized by intraperitoneally injecting pentobarbital sodium (100 mg/kg), and serum samples, liver tissue, and colon tissue were collected. Serum samples and some liver and colon tissue samples were stored at −80°C (Figure 1).

### Detection of serum and liver biochemical indicators

The levels of AST, ALT, TG, TC, LDL-C, and HDL-C in serum were determined using a microplate assay. The levels of SOD in liver tissue were determined using the WST-1 method, MDA levels were determined using the thiobarbituric acid (TBA) method, and GSH-PX levels were determined using an enzymatic assay.

### Enzyme-linked immunosorbent assay

The levels of LPS, IL-6, IL-10, and TNF-α in plasma were determined using an Enzyme-linked immunosorbent assay (ELISA) method.

### Histopathological analysis

Liver and colon tissues were fixed in 10% formalin solution at room temperature for 24 h, then dehydrated with gradient ethanol, embedded in paraffin, stained with hematoxylin & eosin, and the slides were mounted with neutral gum. The frozen liver tissue was embedded in optimal cutting temperature compound, and sliced using a cryoslicer (10 μm). The slices were stained by oil red O staining for evaluating the lipid accumulation in the liver.

### Western blot

The total protein from liver and colon tissues was extracted using a whole protein extraction kit, and the protein content was determined by BCA method. For PPAR-γ, Pck1, Fads1, Acsl4, Plin2, and Occludin, the percentages for the resolving gel and stacking gel, and the transmembrane transfer time were all 8%, 5%, and 90 min, respectively. However, for ZO-1, these values were 6%, 5%, and 150 min, respectively. The proteins were denatured and transferred to PVDF membranes by sodium dodecyl sulfate polyacrylamide gel electrophoresis, and 5% skimmed milk powder was used to block the non-specific binding sites at room temperature for 2 h. Then the membranes were washed three times with TBST, 10 min each time. The primary antibodies PPARγ (1:800), Pck1 (1:800), Fads1 (1:800), Acsl4 (1:3,000), Plin2 (1:800), Occludin (1:800), and ZO-1 (1:2000) were added onto the membranes, incubated at 4 °C overnight. Twenty-four hours later, the membranes were washed with TBST three times, 10 min each time. Then the universal secondary antibody (1:5,000) was incubated with the membranes at room temperature for 1 h, and then the membranes were washed three times with TBST, 10 min each time. Subsequently, an enhanced chemiluminescence reagent was added onto the membranes for color development and detection, and the images were photographed with a gel imager [Analytik Jena Instrument (Beijing)Co., Ltd. (Beijing, China)]. With GAPDH (1:50,000) as an internal reference, grayscale values of the bands were measured using an ImageJ image analysis software (veision 1.8.0), and the ratio of each grayscale value to GAPDH grayscale value was used to express the relative expression level of the proteins.

### 16S rRNA gene sequencing and analysis

The collected fecal samples were stored at −80°C. The 16S rRNA analysis was performed by Novogene Co., Ltd. In Beijing, and total microbial genomic DNA was extracted from mouse fecal samples using a magnetic soil and stool DNA kit (TianGen, China, DP712). The quality and purity of DNA were detected by 1% agarose electrophoresis, and an appropriate amount of sample DNA was put into a centrifuge tube, and diluted with sterile water to 1 ng/μL. The primers 338F (5′-CCTAYGGGRBGCASCAG-3′) and 806R (5′-GACTACNNGGGTATCTAAT-3′) were used to amplify the V3-V4 regions of bacterial 16s rRNA. The PCR amplification system included a 10 ng genomic DNA template, 0.2 μM forward and reverse primers, and 15 μL Phusion^®^ High-Fidelity PCR Master Mix (New England Biolabs). The reaction conditions were the first denaturation at 98°C for 1 min, denaturation at 98°C for 10 s, annealing at 50°C for 30 s, and extension at 72°C for 30 s, with a total of 30 cycles, and finally the samples were stored at 72°C for 5 min. The mixed product was purified by using a universal DNA purification kit (TianGen, China, DP214), and the PCR products were detected by 2% agarose gel electrophoresis. NEB Next ➅ Ultra ™ II FS DNA PCR free Library Prep Kit (NewEngland Biolabs) was used for library construction. The constructed library was quantified by Qubit and Q-PCR, and NovaSeq 6,000 was used for the sequencing on the PE 250 machine after the library was confirmed to be qualified.

### Statistical analysis

The experimental data results were expressed as mean ± standard deviation (mean ± SD). One-way ANOVA analysis was used for statistical comparison between multiple groups of the data, Bonferroni method was used to correct for multiple testing, and post-hoc analysis of the means were conducted with the least significant defference (LSD) test, *p* < 0.05 indicated a significant difference in statistics, and GraphPad Prism 9.5.1 was used for plotting.

## Results

### Effects of Sch B on the liver function of MAFLD mice

**FIGURE 2:**
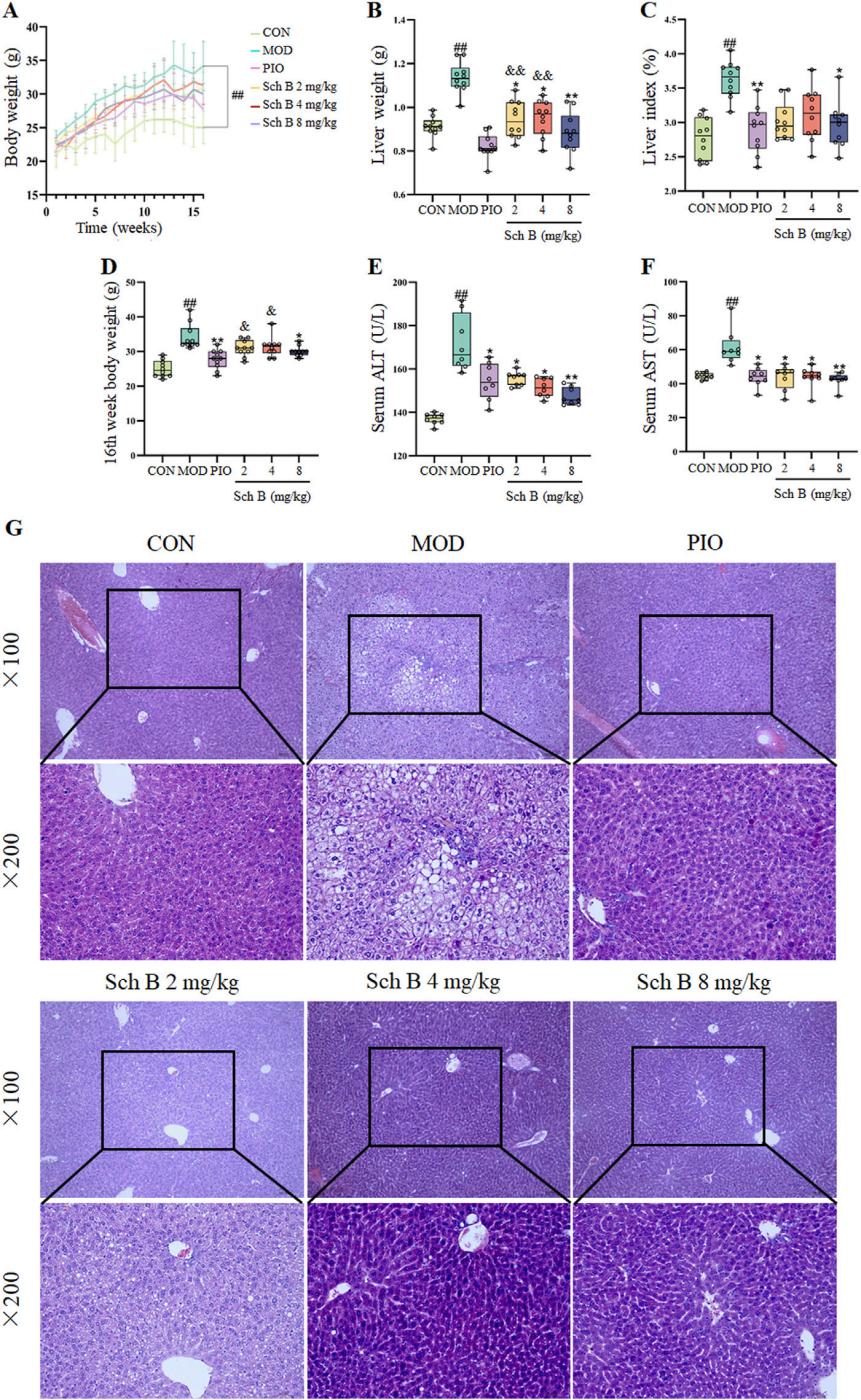
Effects of Sch B on the liver function of MAFLD mice. **(A)**: Body weight; **(B)**: Liver weight; **(C)**: Liver index; **(D)**: 16th week body weight; **(E)**: ALT; **(F)**: AST; **(G)**: H&E staining of liver. All the values were expressed as means ± standard deviation; compared with CON group, ^##^ *p* < 0.01; compared with MOD group, *p < 0.05, **p < 0.01; compared with PIO group, ^&^ *p* < 0.05, ^&&^ *p* < 0.01.

As shown in Figure 2 and Table 1, mice fed with HFD showed a significant increase in the body weight, liver weight, and liver index in the other groups in comparison with those fed with the standard feed in the CON group (*p* < 0.05), while the body weight, liver weight, and liver index were significantly reduced in mice treated with Sch B in comparison with the MOD group (*p* < 0.05), indicating that Sch B could effectively inhibit the weight gain and liver enlargement induced by HFD, but its effect was not as good as pioglitazone (*p* < 0.05). Compared with those in the CON group, the levels of ALT and AST in the serum of mice in the MOD group were significantly increased (*p* < 0.05), while the levels of ALT and AST in the serum showed varying degrees of decrease after the intervention with Sch B (*p* < 0.05), and its function was similar to that of pioglitazone (*p* > 0.05). The H&E staining results showed that the liver lobule structure of mice was regular, without fat accumulation or inflammatory cell infiltration in the liver cells in the CON group, and there were varying degrees of fat vacuoles in the liver tissue, with some hepatocyte necrosis and infiltration of inflammatory cells in the MOD group, while the accumulation of liver fat and infiltration of inflammatory cells in mice were significantly alleviated after the administration of Sch B, indicating that Sch B could alleviate the liver injury caused by HFD.

**TABLE 1:** Effects of Sch B on the liver function of MAFLD mice.

Groups	CON	MOD	PIO	Sch B		
				2 mg/kg	4 mg/kg	8 mg/kg
1st week body weight (g)	21.10 ± 1.97	23.60 ± 0.92	21.90 ± 1.04	22.40 ± 1.43	22.50 ± 0.92	22.40 ± 1.02
16th week body weight (g)	25.00 ± 2.40	34.20 ± 3.65^##^	27.70 ± 2.79^**^	30.90 ± 2.33 ^&^	31.30 ± 2.83 ^&^	30.10 ± 1.45^*^
Liver weight (g)	0.91 ± 0.05	1.14 ± 0.07^##^	0.82 ± 0.06^**^	0.94 ± 0.08^*^^&&^	0.95 ± 0.08^*^^&&^	0.88 ± 0.09^**^
Liver index (%)	2.77 ± 0.30	3.63 ± 0.26^##^	2.90 ± 0.34^**^	3.19 ± 0.38	3.19 ± 0.39	2.97 ± 0.33^*^
ALT (U/L)	136.99 ± 2.58	171.64 ± 12.90^##^	154.37 ± 8.46^*^	155.70 ± 3.16^*^	151.44 ± 4.36^*^	147.40 ± 4.04^**^
AST (U/L)	44.64 ± 1.81	61.62 ± 10.41^##^	43.91 ± 5.58^*^	43.81 ± 7.1^*^	43.85 ± 6.28^*^	42.32 ± 4.24^**^
TC (mmol/L)	1.30 ± 0.11	2.51 ± 0.17^##^	1.67 ± 0.53^*^	1.67 ± 0.22^*^	1.65 ± 0.49^*^	1.46 ± 0.12^**^
TG (mmol/L)	0.51 ± 0.13	1.74 ± 0.43^##^	1.00 ± 0.32^*^	1.12 ± 0.19^*^	0.99 ± 0.12^*^	0.93 ± 0.09^**^
LDL-C (mmol/L)	1.16 ± 0.09	2.52 ± 0.33^##^	1.85 ± 0.28^*^	2.01 ± 0.46^*^	1.95 ± 0.19^*^	1.73 ± 0.29^**^
HDL-C (mmol/L)	4.90 ± 0.53	2.49 ± 0.55^##^	3.40 ± 0.62^*^	3.81 ± 0.49^*^	3.94 ± 0.46^*^	4.12 ± 0.32^**^^&^
SOD (U/mgprot)	50.42 ± 5.71	28.71 ± 9.28^##^	46.72 ± 10.72^**^	45.66 ± 4.30^*^	45.78 ± 2.98^*^	47.60 ± 3.86^**^
MDA (nmol/mgprot)	1.32 ± 0.08	2.15 ± 0.34^##^	1.49 ± 0.12^**^	1.51 ± 0.16^*^	1.50 ± 0.30^*^	1.46 ± 0.24^**^
GSH-PX (µmol/mgprot)	43.36 ± 3.34	24.73 ± 3.72^##^	36.54 ± 1.96^*^	32.85 ± 4.24	38.07 ± 11.15^*^	39.04 ± 4.35^**^
LPS (EU/L)	592.95 ± 40.66	697.49 ± 21.52^##^	634.06 ± 52.85^*^	648.89 ± 14.27	628.90 ± 23.86^*^	613.13 ± 29.87^**^
IL-6 (pg/mL)	41.91 ± 0.83	47.01 ± 30^##^	41.55 ± 1.91^**^	42.59 ± 1.62^*^	42.46 ± 2.79^*^	41.96 ± 1.34^**^
IL-10 (pg/mL)	41.13 ± 1.87	33.34 ± 2.03^##^	39.18 ± 0.66^**^	35.83 ± 2.84 ^&^	35.85 ± 2.13 ^&^	37.84 ± 1.54^*^
TNF-α (pg/mL)	313.6 ± 74.72	398.40 ± 11.94^##^	327.61 ± 22.15^*^	347.21 ± 22.88	334.71 ± 19.6^*^	325.39 ± 19.29^*^

Note: All the values were expressed as means ± standard deviation; compared with CON, group.

^##^ *p* < 0.01; compared with MOD, group, *p < 0.05, **p < 0.01; compared with PIO, group.

^&^ *p* < 0.05.

^&&^ *p* < 0.01.

### Effects of Sch B on lipid metabolism in MAFLD mice

**FIGURE 3:**
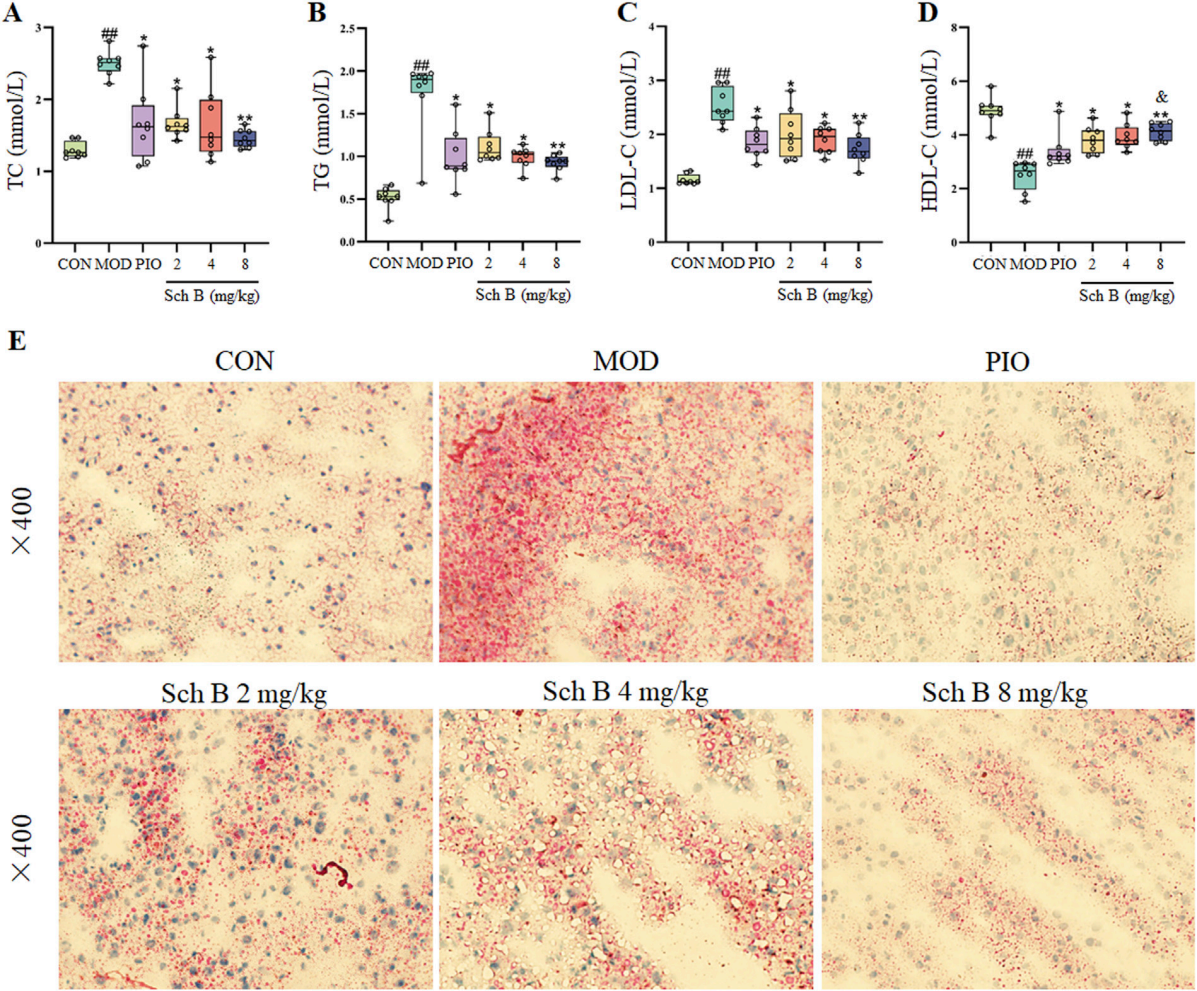
Effects of Sch B on lipid metabolism in MAFLD mice. **(A)**: TC; **(B)**: TG; **(C)**: LDL-C; **(D)**: HDL-C; **(E)**: oil red O staining of liver. All the values were expressed as means ± standard deviation; compared with CON group, ^##^ *p* < 0.01; compared with MOD group, *p < 0.05, **p < 0.01; compared with PIO group, ^&^ *p* < 0.05.

The levels of TC, TG, and LDL-C in the serum of mice were significantly increased (*p* < 0.05), while the level of HDL-C was significantly decreased in the MOD group compared with in the CON group (*p* < 0.05), and the levels of TG, TC, and LDL-C in the serum of mice were significantly decreased (*p* < 0.05), while the level of HDL-C was significantly increased in each Sch B group compared with the MOD group (*p* < 0.05) (Figures 3A–D; Table 1). The oil red O staining showed that there was no significant lipid deposition in the liver tissue of mice in the CON group, while there was a large amount of lipid deposition, with fat droplets occupying most of the liver in the MOD group, and the pathological changes mentioned above were alleviated to varying degrees after the intervention with Sch B (Figure 3E). The above results indicated that the HFD-induced MAFLD model was successfully established, and Sch B could reduce lipid metabolism disorders and liver injury in mice, especially its regulatory effect on HDL-C was even better than that of pioglitazone (Sch B-H vs PIO, *p* < 0.05).

### Effects of Sch B on indicators related to oxidative stress and inflammation

**FIGURE 4:**
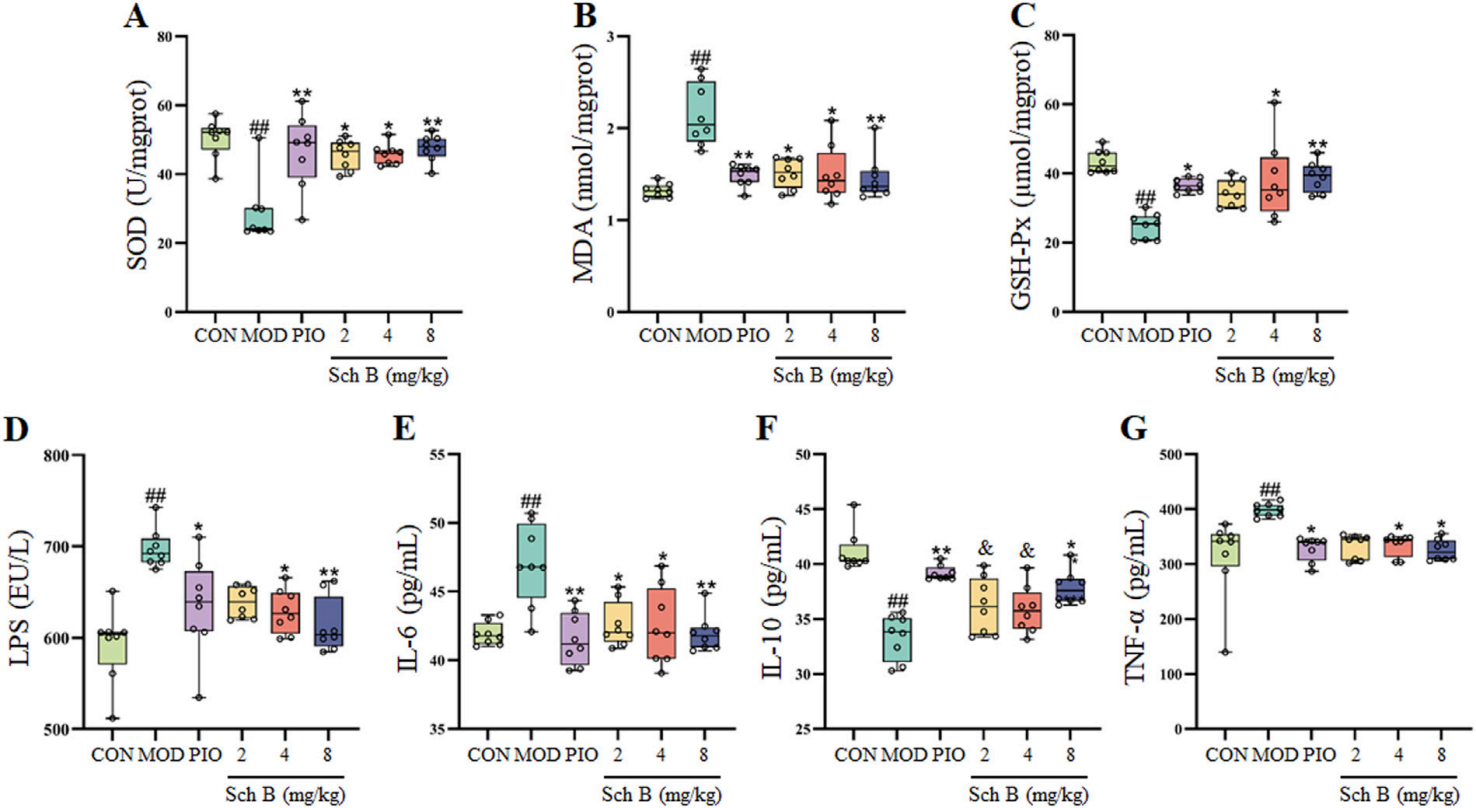
Effects of Sch B on indicators related to oxidative stress and inflammation. **(A)**: SOD; **(B)**: MDA; **(C)**: GSH-Px; **(D)**: LPS; **(E)**: IL-6; **(F)**: IL-10; **(G)**: TNF-α. All the values were expressed as means ± standard deviation; compared with CON group, ^##^ *p* < 0.01; compared with MOD group, *p < 0.05, **p < 0.01; compared with PIO group, ^&^ *p* < 0.05.

Compared with those in the CON group, the contents of MDA in the liver and LPS, TNF-α and IL-6 in the serum of mice were significantly increased (*p* < 0.05), while the activities of SOD and GSH-Px in the liver and the content of IL-10 in the serum were significantly decreased in the MOD group (*p* < 0.05); The activities of SOD and GSH-Px and the content of IL-10 were significantly increased (*p* < 0.05) and the content of MDA was significantly decreased in the liver (*p* < 0.05), and the levels of LPS, TNF-α, and IL-6 in the serum were significantly reduced (*p* < 0.05) in each Sch B-treated group compared with the MOD group (Figure 4; Table 1), indicating that Sch B could alleviate oxidative stress and inflammatory reactions induced by HFD.

### Effects of Sch B on the expression of lipid metabolism-related proteins

**FIGURE 5:**
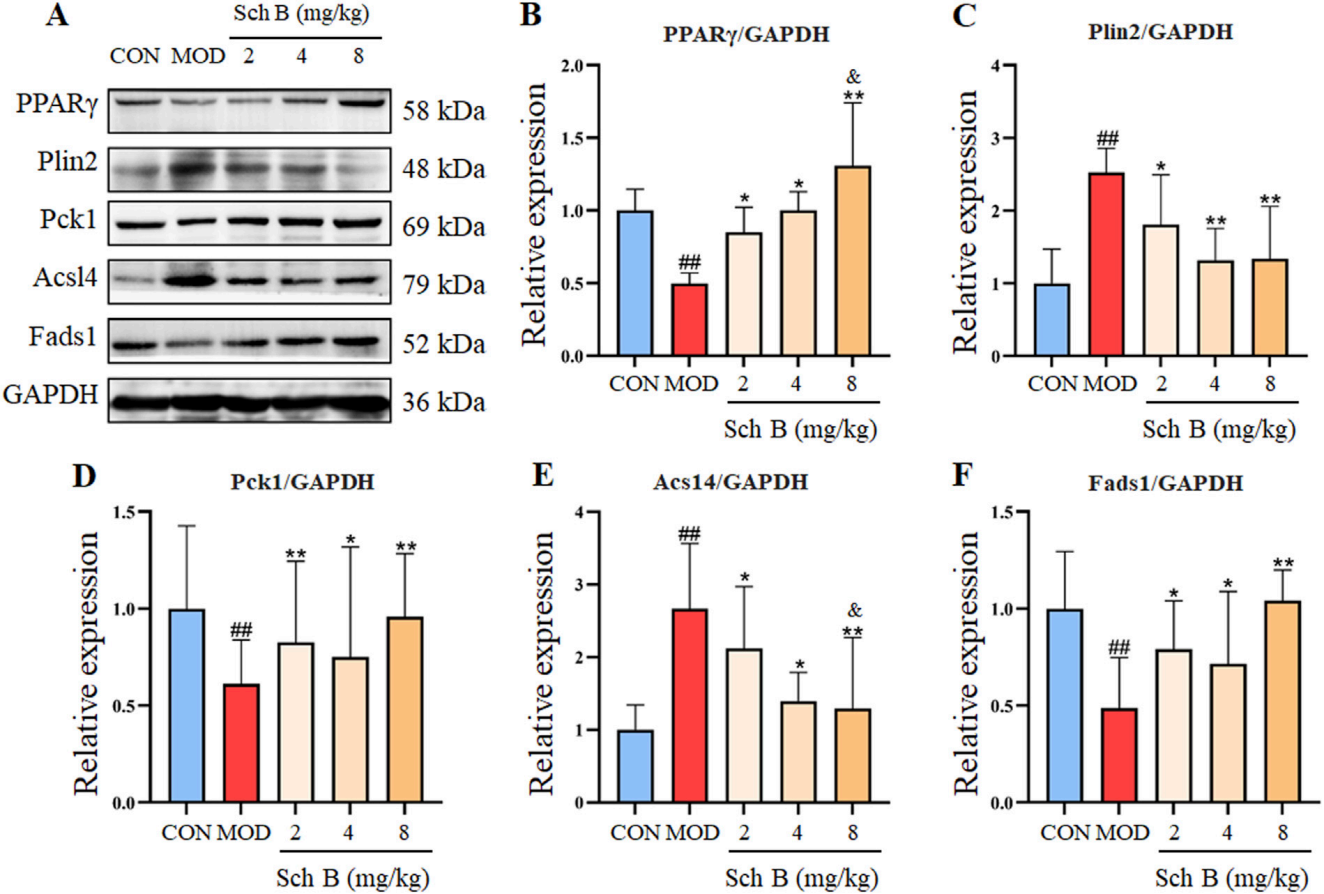
Effects of Sch B on the expression of lipid metabolism-related proteins. **(A)**: Image of PPARγ, Plin2, Pck1, Acsl4, Fads1 and GAPDH protein electrophoresis (Western blot); **(B–F)**: Statistical results of the protein expression. All the values were expressed as means ± standard deviation; compared with CON group, ^##^ *p* < 0.01; compared with MOD group, *p < 0.05, **p < 0.01; compared with Sch B 2 mg/kg group, ^&^ *p* < 0.05.

As shown in Figure 5, compared with those in the CON group, the expression levels of Acsl4 and Plin2 proteins were significantly upregulated in the liver tissue of mice (*p* < 0.05), while the protein expression levels of PPARγ, Fads1, and Pck1 were significantly decreased (*p* < 0.05) in the MOD group; Compared with those in the MOD group, the expression levels of Acsl4 and Plin2 proteins were significantly decreased (*p* < 0.05), while the protein expression levels of PPARγ, Fads1, and Pck1 increased (*p* < 0.05) in Sch B-treated groups, especially in terms of the regulatory effects on PPARγ and Acsl4, there is a clear dose-response relationship (*p* < 0.05).

### Effects of Sch B on the diversity of gut microbiota in MAFLD mice

**FIGURE 6:**
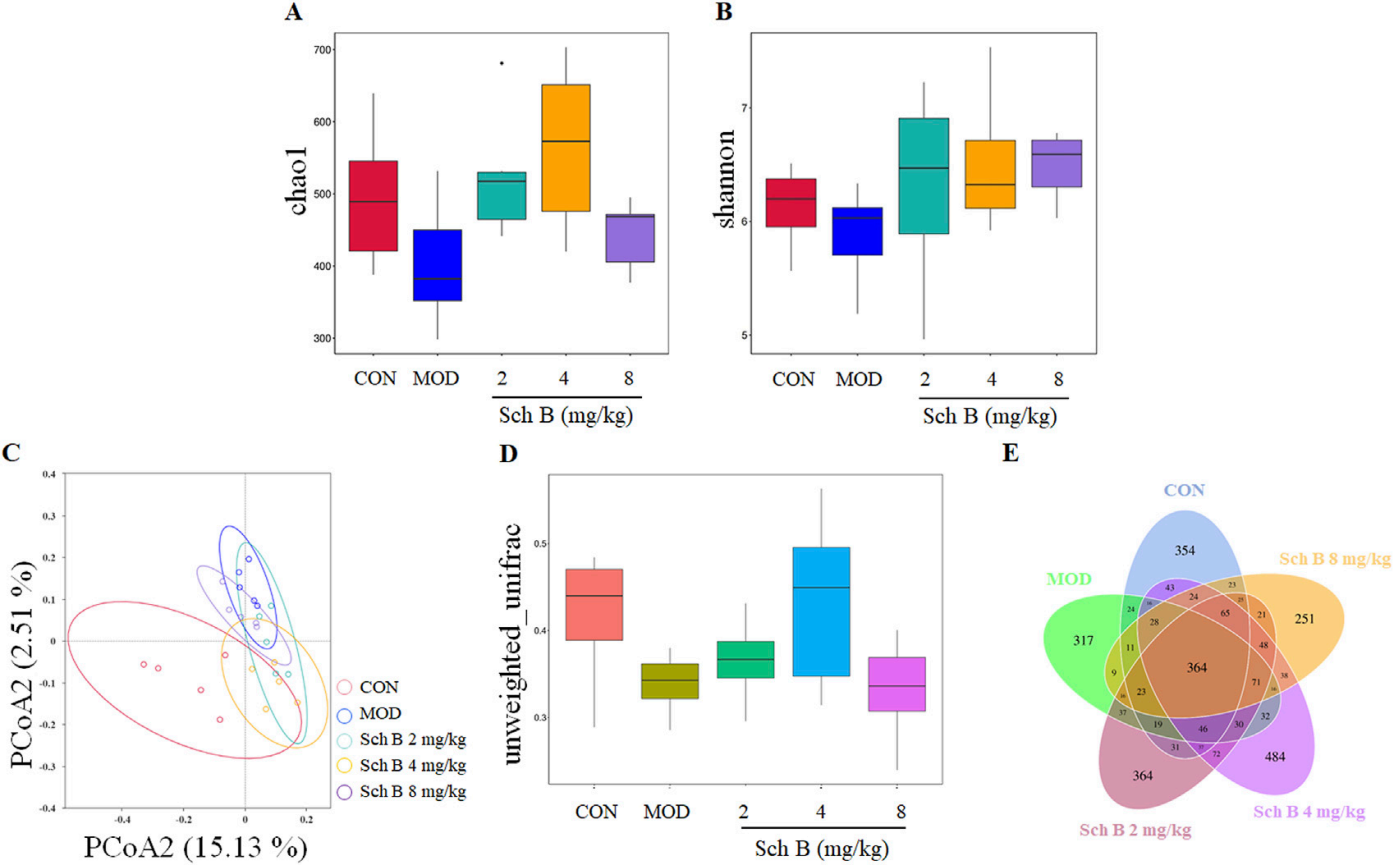
Effects of Sch B on the diversity of gut microbiota in MAFLD mice. **(A)**: Alpha diversity index chao1; **(B)**: shannon; **(C)**: PCoA analysis chart; **(D)**: boxplot analysis; **(E)**: Venn diagram.

In this study, the α-diversity of gut microbiota was evaluated with Chao1 and Shannon indices. Compared with those in the CON group, Chao1 and Shannon indices of the gut microbiota decreased in the MOD group, indicating that MAFLD can reduce the diversity of gut microbiota in mice. Compared with those in the MOD group, the chao1 and Shannon indices increased in Sch B-treated groups (Figures 6A,B), suggesting that Sch B can effectively improve the richness and evenness of gut microbiota in MAFLD mice.

The principal coordinate analysis (PCoA) of β-diversity was used to further analyze differences in gut microbiota. The results showed that the sample points in the CON group and the MOD group were significantly separated, and almost all sample points in the different Sch B-treated groups were closer to those in the CON group (Figure 6C), indicating that their species community composition was more similar. This observation was further supported by boxplot analysis of inter-group β-diversity dissimilarity (Figure 6D). Collectively, these findings suggest that Sch B restores β-diversity of the gut microbiota in MAFLD mice.

The Venn diagram showed significant differences in the number of OTUs among the groups: 1,133 in the CON group, 1,011 in the MOD group, 1,269 in the Sch B-L group, 1,414 in the Sch B-M group, and 1,033 in the Sch B-H group, with 364 in all groups (Figure 6E), indicating that Sch B can improve the gut microbiota disorder caused by HFD in MAFLD mice.

### Effects of Sch B on gut microbiota at phylum and genus levels in MAFLD mice

**FIGURE 7:**
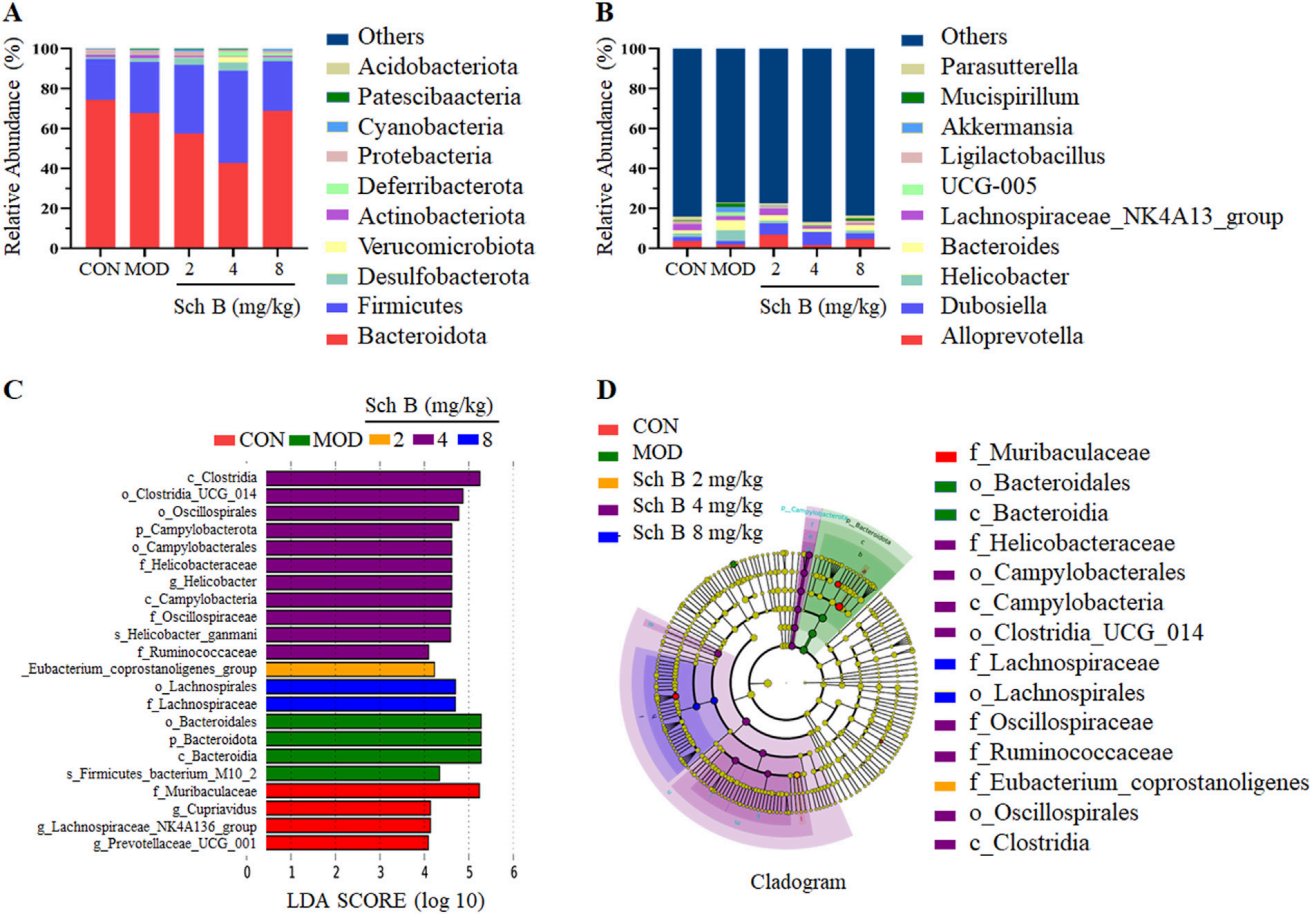
Effects of Sch B on gut microbiota at phylum and genus levels in MAFLD mice. **(A)**: Classification levels of phylum; **(B)**: genus; **(C)**: LEfSe analysis based on LDA value; **(D)**: multi level species hierarchy.

The effect of Sch B on the composition of gut microbiota in MAFLD mice was analyzed using 16S rRNA technology. The analysis at the phylum level showed that *Firmicutes*, *Bacteroidetes*, *Desulfobacterota*, *Verrucomicrobiota*, and *Actinobacteriota* were the dominant bacteria. Compared with that in the CON group, the composition of gut microbiota changed in mice in the MOD group, with an increase in the relative abundance of *Desulfobacterota* and *Firmicutes*, and a decrease in the relative abundance of *Bacteroidetes*, while this trend was effectively reversed in the different Sch B-treated groups (Figure 7A).

The analysis at the genus level showed that *Alloprevotella*, *Dubosiella*, *Helicobacter*, *Bacteroides*, and *Lachnospiraceae*_NK4A136_group were the main dominant bacteria. The abundance of *Helicobacter*, *Bacteroides*, *Mucispirillum*, and UCG-005 was higher, while the abundance of *AlloPrevotella*, *Dubosiella*, and *Lachnospiraceae*_NK4A136_group was lower in the MOD group compared with the CON group. The abundance of two beneficial bacteria, *AlloPrevotella* and *Lachnospiraceae*_NK4A136_group, increased, while the abundance of harmful bacteria, *Helicobacter*, decreased in the Sch B-treated groups compared with the MOD group. The above results indicate that Sch B can improve the gut microbiota of MAFLD mice fed with HFD (Figure 7B).

LEfSe was used for the differential analysis at all levels simultaneously, and a LDA value of 4 was used to screen iconic species with significantly differences among groups. The results showed that a total of 22 significantly iconic species were selected from these 5 groups, among which there were 4 in the CON group, 4 in the MOD group, 1 in the Sch B-L group, 11 in the Sch B-M group, and 2 in the Sch B-H group (Figures 7C,D).

**FIGURE 8:**
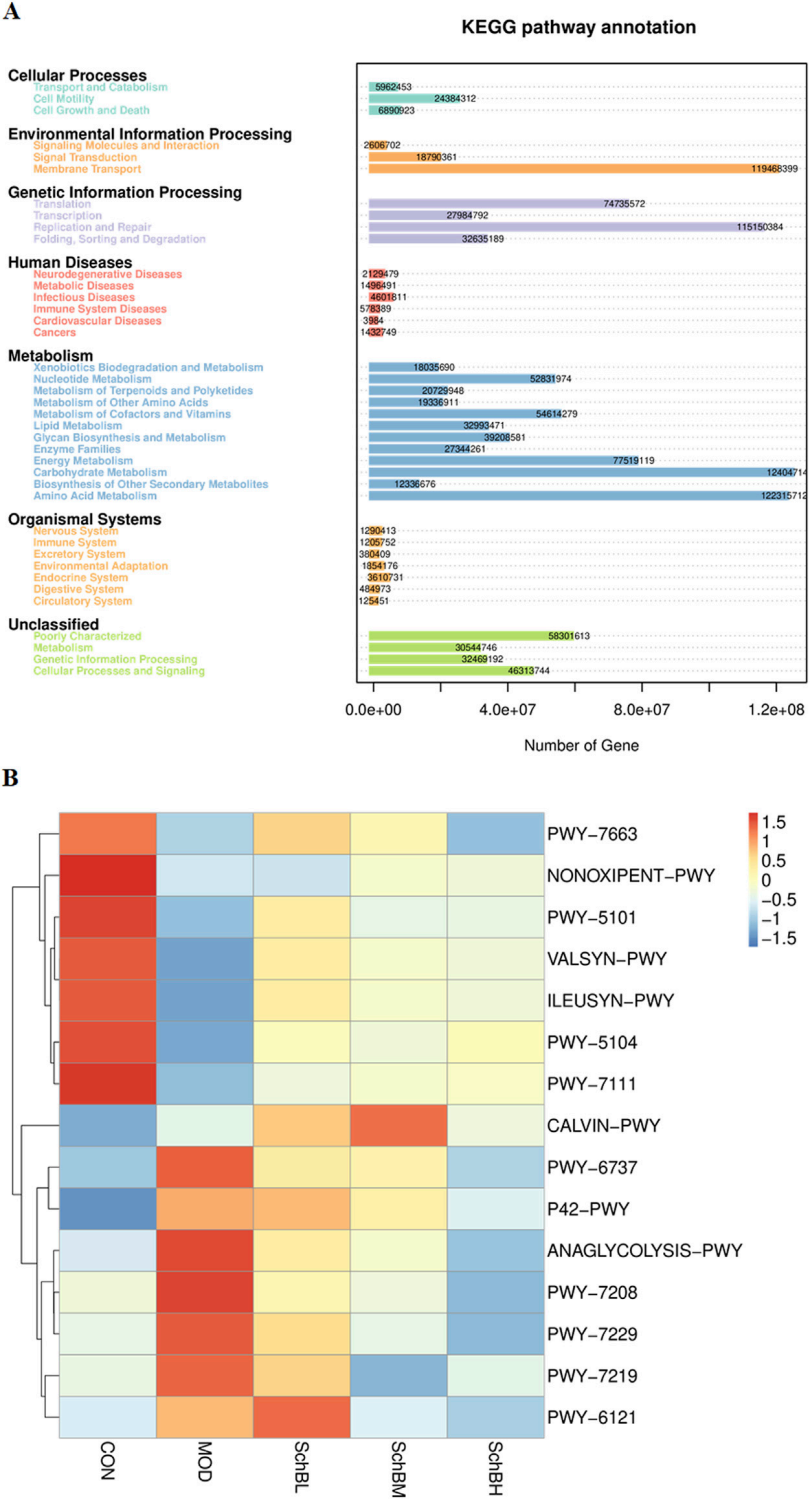
Predicted KEGG secondary functional pathways **(A)** and PICRUSt2 functional annotation cluster heatmap **(B)**.

Pathway enrichment analysis of the gut microbiota data was performed using the KEGG database. The prediction revealed that the secondary functional classifications were predominantly centered on Amino Acid Metabolism, Carbohydrate Metabolism, Replication and Repair, and Membrane Transport (Figure 8A). To further investigate the impact of Sch B on the gut microbiota, functional prediction of the samples was conducted using PICRUSt2 based on the KEGG Pathway database. The prediction results demonstrated significant differences in metabolic pathways between the control group and the model group. Notably, the Sch B treatment groups exhibited a shift toward the pattern observed in the control group. Specifically, the correlation for PWY-5104 (L-isoleucine biosynthesis IV) and PWY-7111 (pyruvate fermentation to isobutanol) was significantly increased. Conversely, the correlation for PWY-7208 (superpathway of pyrimidine nucleobases salvage) and PWY-7229 (superpathway of adenosine nucleotides *de novo* biosynthesis I) was significantly decreased (Figure 8B).

### Effects of Sch B on the intestinal homeostasis in MAFLD mice

**FIGURE 9:**
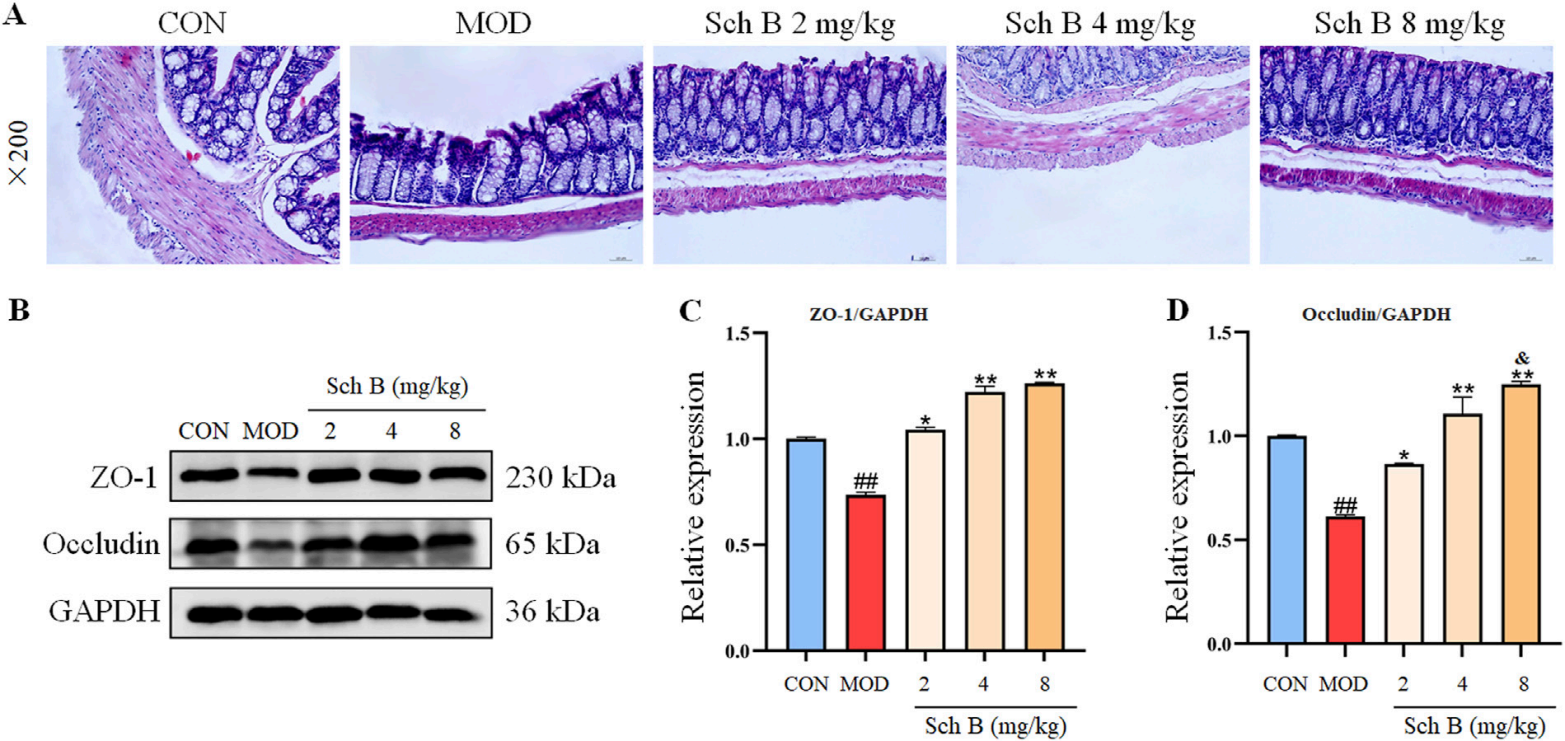
Effects of Sch B on the intestinal homeostasis in MAFLD mice. **(A)**: H&E staining of colon; **(B)**: image of ZO-1, Occludin and GAPDH protein electrophoresis (Western blot); **(C,D)**: Statistical results of the protein expression. All the values were expressed as means ± standard deviation; compared with CON group, ^##^ *p* < 0.01; compared with MOD group, *p < 0.05, **p < 0.01; compared with Sch B 2 mg/kg group, ^&^ *p* < 0.05.

A HFD can induce lipopolysaccharide translocation, leading to intestinal inflammation and damage to the gut barrier. The HE staining results showed that the colon villi of the MOD group mice were sparse and shortened, the glandular hyperplasia was inactive, and the intrinsic muscle layer became thinner, indicating that the HFD could cause a severe damage to the gut barrier, while Sch B could significantly alleviate the above changes. The Western blot results showed that the expression levels of tight junction proteins ZO-1 and Occludin were significantly reduced in the MOD group (*p* < 0.05), while those of the two proteins were significantly upregulated in the Sch B-treated groups (*p* < 0.05), especially in terms of the regulatory effects on Occludin, there is a clear dose-response relationship (*p* < 0.05) (Figure 9), suggesting that Sch B may maintain and repair the integrity of gut barrier function by restoring the level of tight junction proteins.

## Discussion

A HFD can lead to an excessive fat accumulation in the liver and other tissues, and then induce obesity and metabolic diseases, accompanied by the occurrence of MAFLD (Lian et al., 2020). Our research results showed that the treatment with Sch B could not only significantly reduce the body weight and liver weight, but also alleviate the steatosis and inflammatory infiltration in the liver of MAFLD mice. In addition, Sch B showed significant effects in modulating blood lipid levels, and alleviating the oxidative stress and inflammatory reactions in MAFLD mice.

PPARγ, as a key factor regulating lipid metabolism, forms an active heterodimer when paired with nuclear retinoid X receptors (RXRs), plays an important role in regulating adipocyte differentiation, adipogenesis, and glucose metabolism (Zhu et al., 1993; Samuel and Shulman, 2018). Plin2 is a protein associated with intracellular lipid droplet metabolism, playing a critical role in fatty acid uptake, lipid droplet formation, and lipid storage (Conte et al., 2016). It has been shown that the normal expression of Plin2 leads to increased obesity, while the deletion of Plin2 gene induces an obesity resistance phenotype in wild-type (WT) mice fed with a HFD to prevent the weight gain caused by the HFD (McManaman et al., 2013). Pck1 is a regulator of the energy metabolism and gluconeogenesis in the liver, and its imbalance is related to metabolic diseases, such as diabetes, obesity and insulin resistance, while there is also a certain correlation between PPARγ and Pck1 (Beale et al., 2007; Tuo et al., 2019). Silencing gene Pck1 in the liver can lower the blood glucose, and alleviate the insulin sensitivity and dyslipidemia in db/db mice (Gómez-Valadés et al., 2008). ACSL4 is a key metabolic isoenzyme for polyunsaturated fatty acids, localized in peroxisomes and the endoplasmic reticulum. As an essential metabolic enzyme mediating fatty acid activation, it can enhance or suppress fatty acid activation through transcriptional regulation, thereby promoting or alleviating hepatic steatosis (Kan et al., 2015; Sui et al., 2024; Doll et al., 2020; Wurst et al., 2017). Fads1, also known as δ −5 desaturase (D5D), is one of rate-limiting enzymes in the pathway of polyunsaturated fatty acid desaturation (Athinarayanan et al., 2020). The decreased expression of Fads1 is associated with the increased total lipid content in the liver, as well as obesity, MAFLD, and other metabolic disorders (Araya et al., 2010; Wang et al., 2015; Murakami et al., 2008). In this study, it was found that the upregulation of PPARγ induced by the administration of Sch B could upregulate the expression of downstream proteins Pck1 and Fads1, and downregulate the expression of Plin2 and Acsl4, and the administration of Sch B could alleviate the MAFLD induced by HFD by regulating lipid metabolism, enhancing energy metabolism, and regulating lipid droplet degradation. These findings indicate that Sch B can regulate lipid metabolism and lipid droplet degradation by PPARγ, Plin2, Pck1, ACSL4 and Fads1 signaling pathway, while improving insulin resistance, thereby alleviating hepatic lipid accumulation caused by MAFLD.

Changes in the gut microbiota environment play an important role in the progression of MAFLD. Both beneficial and harmful gut microbiota metabolites can be transported directly to the liver through the portal vein, where they participate in many biological processes including the production of energy, detoxification and synthesis (Barber et al., 2023; Alghamdi et al., 2024; Abenavoli et al., 2023). The 16S rRNA sequencing results in this study showed that Sch B could improve the diversity of gut microbiota in MAFLD mice and also alter the composition of the microbiota by increasing the abundance of beneficial bacteria and reducing the growth of harmful bacteria. Studies have shown that an increased *Firmicutes-to-Bacteroidetes* (F/B) ratio is closely associated with obesity. Since both *Firmicutes* and *Bacteroidetes* are involved in energy harvest, an imbalance in their proportions may lead to excessive energy absorption (Fontané et al., 2018; Tenorio-Jiménez et al., 2020). It was found in this study that the composition of the gut microbiota was significantly altered in mice feed with HFD, with an increase in the abundance of *Firmicutes* and a decrease in the abundance of *Bacteroidetes*, resulting in an increase in the F/B ratio, while the F/B ratio showed a decreasing trend after the treatment with Sch B. The composition of gut microbiota in MAFLD mice also changed at the genus level, including changes in both beneficial and harmful bacteria, and these changes in microbiota may also be the key to the treatment of MAFLD.

It has been demonstrated that *Alloprevotella* is negatively correlated with body weight, fat weight, serum TG, TC, and HDL-C. *Alloprevotella* has been shown to produce various short-chain fatty acid metabolites (Huang et al., 2023; Zhao et al., 2021) and the short-chain fatty acids produced can increase energy expenditure, reduce weight, and decrease liver TG accumulation by activating the PPAR signaling pathway, thereby regulating lipid metabolism and improving obesity (Ai et al., 2022). Moreover, *Alloprevotella* is negatively correlated with fasting blood glucose, insulin resistance index, and area under the GTT curve, which can enhance satiety and maintain glucose homeostasis by producing glucagon-like peptide-1 (GLP-1) (Li et al., 2021; Ni et al., 2023). It has been shown that *Bacteroides* can effectively reduce the weight gain induced by high-fat diet in mice and regulate the expression of lipid metabolism-related genes in white adipose tissue (Liu et al., 2017). *Bacteroides* can significantly improve liver steatosis and TG accumulation by repairing intestinal barrier function and reducing lipopolysaccharide translocation (Sangineto et al., 2022). Furthermore, its derived sphingolipids can be directly transferred from the microbiome to the host colon and liver, further alleviating the diet-induced hepatic steatosis (Le et al., 2022).

*The Lachnospiraceae*_NK4A136_group is recognized as a probiotic strain capable of producing short-chain fatty acids with antimicrobial activity against pathogens and an anti-inflammatory molecule, and the butyric acid transformed from it helps maintain intestinal barrier integrity in mice, showing a negative correlation with intestinal permeability; Butyrate is one of the main short chain fatty acids in the microbiota because it can enhance epithelial barrier integrity and inhibit inflammation (Breitrück et al., 2021; Wu et al., 2021). Studies have shown that *Dubosiella* is a probiotic, and the increase of its relative abundance has been proved to have potential benefits for metabolic diseases such as type 2 diabetes mellitus and steatohepatitis, mainly through enhancing glucose tolerance, inhibiting inflammation, and improving insulin sensitivity and blood lipid levels (Li et al., 2022; Zheng et al., 2023). In addition, *Dubosiella* is negatively correlated with the mRNA expression levels of pro-inflammatory cytokines IL-1β, IL-6, and TNF-α. On the contrary, *Dubosiella* is positively correlated with IL-10, an anti-inflammatory factor (Wan et al., 2021). The chronic gastritis induced by *Helicobacter*, as a common pathogenic bacterium in the stomach, can cause a low-grade systemic chronic inflammation, and the local inflammation of gastric mucosa can lead to the release of pro-inflammatory factors and then the increase of intestinal permeability and the production of endotoxins, which then enter the liver through the portal vein, causing the inflammation and fat accumulation in the liver (Martin-Nuñez et al., 2021; Mavilia-Scranton et al., 2023; Tang et al., 2022).

The gut barrier function is one of the most fundamental functions of epithelial cells and the basis for ensuring the integrity of the cytoskeleton and membrane proteins, and these cytoskeleton and membrane proteins can form and regulate tight junction complexes among cells (Hull and Staehelin, 1979). Lipopolysaccharides (LPS) are an important component of the intestinal microbiota cell wall. When the intestinal mucosal barrier is disrupted, LPS from the gut microbiota can be released into the bloodstream, exacerbating the body’s inflammatory response (Cândido et al., 2018). The destruction of the gut barrier can lead to an increase in the permeability of the intestine to bacteria and their derivatives, causing immune system disorders and inflammatory reactions, all of which can lead to the progression of MAFLD (Martín-Mateos and Albillos, 2021). ZO-1 and Occludin are both tight junction proteins that play a crucial role in maintaining the integrity of the gut barrier, and their elevated expression can reduce the permeability of intestines, restore the integrity of the gut barrier, and prevent endotoxins or bacteria from entering the blood circulation, restoring the ecosystem of the intestinal microbiota. The results of this study indicate that Sch B can upregulate the expression of tight junction proteins ZO-1 and Occludin, restoring the intestinal damage caused by LPS and protecting the integrity of the gut barrier.

## Conclusion

**FIGURE 10:**
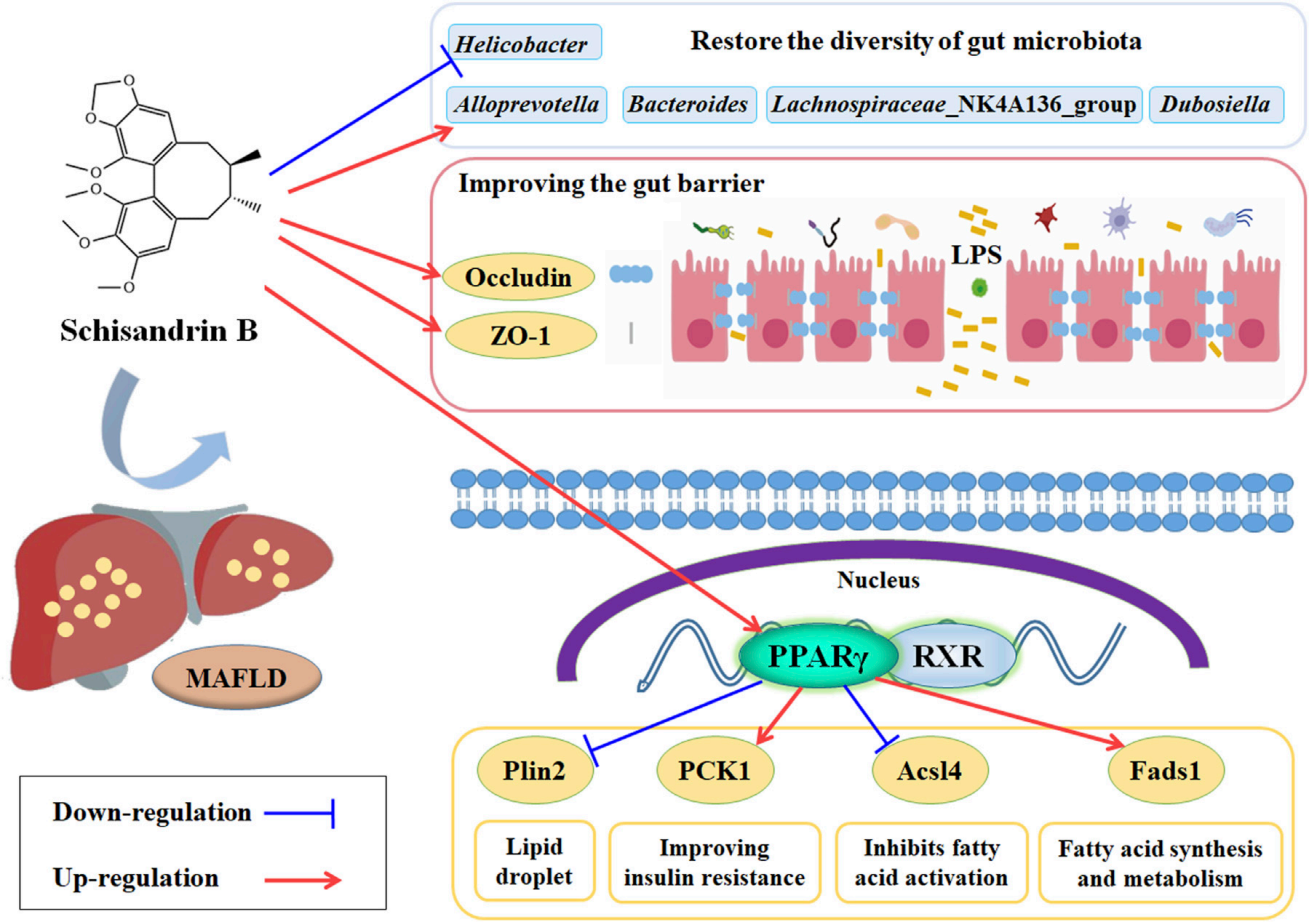
Potential mechanism of Sch B alleviate the MAFLD mice induced by HFD.

Sch B can alleviate the liver injury, the lipid disorders and hepatic steatosis, and the oxidative stress and inflammatory response in MAFLD mice fed with HFD. It may alleviate MAFLD through the mechanism of regulating the PPARγ, Plin2, Pck1, Acl4 and Feds1 signaling pathway and increasing the expression of ZO-1 and Occludin proteins to restore the balance of gut microbiota, increasing the abundance of beneficial bacteria such as *AlloPrevotella* and *Lachnospiraceae*_NK4A136_group to ameliorate the gut barrier injury and maintain the gut barrier integrity in MAFLD mice (Figure 10).

## Data Availability

The data presented in the study are deposited in the NCBI repository, accession number PRJNA1291467.

## References

[B1] Abenavoli L. Scarlata G. G. M. Scarpellini E. Boccuto L. Spagnuolo R. Tilocca B. (2023). Metabolic-dysfunction-associated fatty liver disease and gut microbiota: from fatty liver to dysmetabolic syndrome. Med. Kaunas. 59, 594. 10.3390/medicina59030594 PMC10054528 36984595

[B2] Ai Z. L. Zhang X. Ge W. Zhong Y. B. Wang H. Y. Zuo Z. Y. (2022). Salvia miltiorrhiza extract may exert an anti-obesity effect in rats with high-fat diet-induced obesity by modulating gut microbiome and lipid metabolism. World J. Gastroenterol. 28, 6131–6156. 10.3748/wjg.v28.i43.6131 36483153 PMC9724488

[B3] Alghamdi W. Mosli M. Alqahtani S. A. (2024). Gut microbiota in MAFLD: therapeutic and diagnostic implications. Ther. Adv. Endocrinol. Metab. 15, 20420188241242937. 10.1177/20420188241242937 38628492 PMC11020731

[B4] Araya J. Rodrigo R. Pettinelli P. Araya A. V. Poniachik J. Videla L. A. (2010). Decreased liver fatty acid delta-6 and delta-5 desaturase activity in obese patients. Obes. (Silver Spring) 18, 1460–1463. 10.1038/oby.2009.379 19875987

[B5] Athinarayanan S. Fan Y. Y. Wang X. Callaway E. Cai D. Chalasani N. (2020). Fatty acid desaturase 1 influences hepatic lipid homeostasis by modulating the PPARα-FGF21 axis. Hepatol. Commun. 5, 461–477. 10.1002/hep4.1629 33681679 PMC7917273

[B6] Barber T. M. Hanson P. Weickert M. O. (2023). Metabolic-associated fatty liver disease and the gut microbiota. Endocrinol. Metab. Clin. North Am. 52, 485–496. 10.1016/j.ecl.2023.01.004 37495339

[B7] Beale E. G. Harvey B. J. Forest C. (2007). PCK1 and PCK2 as candidate diabetes and obesity genes. Cell Biochem. Biophys. 48, 89–95. 10.1007/s12013-007-0025-6 17709878

[B8] Breitrück A. Weigel M. Hofrichter J. Sempert K. Kerkhoff C. Mohebali N. (2021). Smectite as a preventive oral treatment to reduce clinical symptoms of DSS induced colitis in Balb/c mice. Int. J. Mol. Sci. 22, 8699. 10.3390/ijms22168699 34445403 PMC8395406

[B9] Cândido F. G. Valente F. X. Grześkowiak Ł. M. Moreira A. P. B. Rocha D. M. U. P. Alfenas R. C. G. (2018). Impact of dietary fat on gut microbiota and low-grade systemic inflammation: mechanisms and clinical implications on obesity. Int. J. Food Sci. Nutr. 69, 125–143. 10.1080/09637486.2017.1343286 28675945

[B10] Conte M. Franceschi C. Sandri M. Salvioli S. (2016). Perilipin 2 and age-related metabolic diseases: a new perspective. Trends Endocrinol. Metab. 27, 893–903. 10.1016/j.tem.2016.09.001 27659144

[B11] Doll S. Proneth B. Tyurina Y. Y. Panzilius E. Kobayashi S. Ingold I. (2020). A new definition for metabolic dysfunction-associated fatty liver disease: an international expert consensus statement. J. Hepatol. 73, 202–209. 10.1016/j.jhep.2020.03.039 32278004

[B48] Eslam M. Newsome P. N. Sarin S. K. Anstee Q. M. Targher G. Romero-Gomez M. (2020). A new definition for metabolic dysfunction-associated fatty liver disease: an international expert consensus statement. J. Hepatol. 73 (1), 202–209. 10.1016/j.jhep.2020.03.039 32278004

[B12] Feng Y. B. Li H. Chen C. Lin H. Xu G. Y. Li H. (2021). Study on the hepatoprotection of Schisandra chinensis caulis polysaccharides in nonalcoholic fatty liver disease in rats based on metabolomics. Front. Pharmacol. 12, 727636. 10.3389/fphar.2021.727636 34621168 PMC8490749

[B13] Fontané L. Benaiges D. Goday A. Llauradó G. Pedro-Botet J. (2018). Influence of the microbiota and probiotics in obesity. Clin. Investig. Arterioscler. 30, 271–279. English, Spanish. 10.1016/j.arteri.2018.03.004 29804899

[B14] Gómez-Valadés A. G. Méndez-Lucas A. Vidal-Alabró A. Blasco F. X. Chillon M. Bartrons R. (2008). Pck1 gene silencing in the liver improves glycemia control, insulin sensitivity, and dyslipidemia in Db/db mice. Diabetes 57, 2199–2210. 10.2337/db07-1087 18443203 PMC2494684

[B15] Huang Y. H. Wang Z. J. Ye B. Ma J. H. Ji S. L. Sheng W. (2023). Sodium butyrate ameliorates diabetic retinopathy in mice via the regulation of gut microbiota and related short-chain fatty acids. J. Transl. Med. 21, 451. 10.1186/s12967-023-04259-4 37420234 PMC10329333

[B16] Hull B. E. Staehelin L. A. (1979). The terminal web. A reevaluation of its structure and function. J. Cell Biol. 81, 67–82. 10.1083/jcb.81.1.67 573268 PMC2111517

[B17] Ji Y. Yin Y. Sun L. Zhang W. (2020). The molecular and mechanistic insights based on gut-liver axis: nutritional target for non-alcoholic fatty liver disease (NAFLD) improvement. Int. J. Mol. Sci. 21, 3066. 10.3390/ijms21093066 32357561 PMC7247681

[B18] Kan C. F. Singh A. B. Dong B. Shende V. R. Liu J. (2015). PPARδ activation induces hepatic long-chain acyl-CoA synthetase 4 expression *in vivo* and *in vitro* . Biochim. Biophys. Acta 1851, 577–587. 10.1016/j.bbalip.2015.01.008 25645621 PMC5292870

[B19] Le H. H. Lee M. T. Besler K. R. Johnson E. L. (2022). Host hepatic metabolism is modulated by gut microbiota-derived sphingolipids. Cell Host Microbe 30, 798–808.e7. 10.1016/j.chom.2022.05.002 35623356 PMC9187616

[B20] Li B. Y. Mao Q. Q. Xiong R. G. Zhou D. D. Huang S. Y. Saimaiti A. (2022). Preventive effects of different black and dark teas on obesity and non-alcoholic fatty liver disease and modulate gut microbiota in high-fat diet fed mice. Foods 11, 3457. 10.3390/foods11213457 36360069 PMC9658379

[B21] Li S. You J. M. Wang Z. R. Liu Y. Wang B. Du M. (2021). Curcumin alleviates high-fat diet-induced hepatic steatosis and obesity in association with modulation of gut microbiota in mice. Food Res. Int. 143, 110270. Epub 2021 Mar 9. 10.1016/j.foodres.2021.110270 33992371

[B22] Lian C. Y. Zhai Z. Z. Li Z. F. Wang L. (2020). High fat diet-triggered non-alcoholic fatty liver disease: a review of proposed mechanisms. Chem. Biol. Interact. 330, 109199. 10.1016/j.cbi.2020.109199 32805210

[B23] Liu H. Li H. Yuan R. S. Sun J. H. Chen J. G. Wang C. M. (2016). Effect of Schisandra chinensis lignans on hyperlipidemia in C57BL/6 mice. Food Sci. 37, 218–221. (Chinese). 10.7506/spkx1002-6630-201611038

[B24] Liu R. X. Hong J. Xu X. Q. Feng Q. Zhang D. Y. Gu Y. Y. (2017). Gut microbiome and serum metabolome alterations in obesity and after weight-loss intervention. Nat. Med. 23, 859–868. 10.1038/nm.4358 28628112

[B25] Martín-Mateos R. Albillos A. (2021). The role of the gut-liver axis in metabolic dysfunction-associated fatty liver disease. Front. Immunol. 12, 660179. 10.3389/fimmu.2021.660179 33936094 PMC8085382

[B26] Martin-Nuñez G. M. Cornejo-Pareja I. Clemente-Postigo M. Tinahones F. J. (2021). Gut microbiota: the missing link between Helicobacter pylori infection and metabolic disorders? Front. Endocrinol. (Lausanne) 12, 639856. 10.3389/fendo.2021.639856 34220702 PMC8247771

[B27] Mavilia-Scranton M. G. Wu G. Y. Dharan M. (2023). Impact of Helicobacter pylori infection on the pathogenesis and management of nonalcoholic fatty liver disease. J. Clin. Transl. Hepatol. 11, 670–674. 10.14218/JCTH.2022.00362 36969902 PMC10037521

[B28] McManaman J. L. Bales E. S. Orlicky D. J. Jackman M. MacLean P. S. Cain S. (2013). Perilipin-2-null mice are protected against diet-induced obesity, adipose inflammation, and fatty liver disease. J. Lipid Res. 54, 1346–1359. 10.1194/jlr.M035063 23402988 PMC3622329

[B29] Murakami K. Sasaki S. Takahashi Y. Uenishi K. Watanabe T. Kohri T. (2008). Lower estimates of delta-5 desaturase and elongase activity are related to adverse profiles for several metabolic risk factors in young Japanese women. Nutr. Res. 28, 816–824. 10.1016/j.nutres.2008.08.009 19083494

[B30] Ni J. Shangguan Y. C. Jiang L. L. He C. B. Ma Y. Xiong H. J. (2023). Pomelo peel dietary fiber ameliorates alterations in obesity-related features and gut microbiota dysbiosis in mice fed on a high-fat diet. Food Chem. X 20, 100993. 10.1016/j.fochx.2023.100993 38144811 PMC10740135

[B31] Pipitone R. M. Ciccioli C. Infantino G. La M. C. Parisi S. Tulone A. (2023). MAFLD: a multisystem disease. Ther. Adv. Endocrinol. Metab. 14, 20420188221145549. 10.1177/20420188221145549 36726391 PMC9885036

[B32] Samuel V. T. Shulman G. I. (2018). Nonalcoholic fatty liver disease as a nexus of metabolic and hepatic diseases. Cell Metab. 27, 22–41. 10.1016/j.cmet.2017.08.002 28867301 PMC5762395

[B33] Sangineto M. Grander C. Grabherr F. Mayr L. Enrich B. Schwärzler J. (2022). Recovery of Bacteroides thetaiotaomicron ameliorates hepatic steatosis in experimental alcohol-related liver disease. Gut Microbes 14, 2089006. 10.1080/19490976.2022.2089006 35786161 PMC9255095

[B34] Sui Y. Geng X. Wang Z. Zhang J. Yang Y. Meng Z. (2024). Targeting the regulation of iron homeostasis as a potential therapeutic strategy for nonalcoholic fatty liver disease. Metabolism 157, 155953. 10.1016/j.metabol.2024.155953 38885833

[B35] Sun J. H. Lin H. Wang K. Zeng Q. C. Li H. Wang C. M. (2022). Study of Schisandra chinensis in treating metabolic associated fatty liver disease based on network pharmacology and molecular docking technology. J. Beihua Univ. Nat. Sci. 23, 768–774. (Chinese). 10.11713/j.issn.1009-4822.2022.06.013

[B36] Sun J. H. Liu X. Cong L. X. Li H. Zhang C. Y. Chen J. G. (2017). Metabolomics study of the therapeutic mechanism of Schisandra chinensis lignans in diet-induced hyperlipidemia mice. Lipids Health Dis. 16, 145. 10.1186/s12944-017-0533-3 28764799 PMC5537938

[B37] Tang W. Yuan M. Li Z. Lin Q. Zhen Y. Li Z. (2022). Polyphenol-rich liupao tea extract prevents high-fat diet-induced MAFLD by modulating the gut microbiota. Nutrients 14, 4930. 10.3390/nu14224930 36432617 PMC9697786

[B38] Tenorio-Jiménez C. Martínez-Ramírez M. J. Gil Á. Gómez-Llorente C. (2020). Effects of probiotics on metabolic syndrome: a systematic review of randomized clinical trials. Nutrients 12, 124. 10.3390/nu12010124 31906372 PMC7019472

[B39] Tuo L. Xiang J. Pan X. Hu J. Tang H. Liang L. (2019). PCK1 negatively regulates cell cycle progression and hepatoma cell proliferation via the AMPK/p27Kip1 axis. J. Exp. Clin. Cancer Res. 38, 50. 10.1186/s13046-019-1029-y 30717766 PMC6360696

[B40] Wan F. Han H. Zhong R. Q. Wang M. Y. Tang S. L. Zhang S. F. (2021). Dihydroquercetin supplement alleviates colonic inflammation potentially through improved gut microbiota community in mice. Food Funct. 12, 11420–11434. 10.1039/d1fo01422f 34673859

[B41] Wang L. Athinarayanan S. Jiang G. Chalasani N. Zhang M. Liu W. (2015). Fatty acid desaturase 1 gene polymorphisms control human hepatic lipid composition. Hepatology 61, 119–128. 10.1002/hep.27373 25123259 PMC4280302

[B42] Wu H. Chen Q. Liu J. Chen X. Luo H. Ye Z. (2021). Microbiome analysis reveals gut microbiota alteration in mice with the effect of matrine. Microb. Pathog. 156, 104926. 10.1016/j.micpath.2021.104926 33964419

[B43] Wu Z. Tian E. Chen Y. Dong Z. Peng Q. (2023). Gut microbiota and its roles in the pathogenesis and therapy of endocrine system diseases. Microbiol. Res. 268, 127291. 10.1016/j.micres.2022.127291 36542917

[B44] Wurst W. Schick J. A. Kagan V. E. Angeli J. P. Conrad M. Ingold I. (2017). ACSL4 dictates ferroptosis sensitivity by shaping cellular lipid composition. Nat. Chem. Biol. 13, 91–98. 10.1038/nchembio.2239 27842070 PMC5610546

[B45] Zhao Q. Y. Hou D. Z. Fu Y. X. Xue Y. Guan X. Shen Q. (2021). Adzuki bean alleviates obesity and insulin resistance induced by a high-fat diet and modulates gut microbiota in mice. Nutrients 13, 3240. 10.3390/nu13093240 34579118 PMC8466346

[B46] Zheng X. X. Li D. X. Li Y. T. Chen Y. L. Zhao Y. L. Ji S. (2023). Mulberry leaf water extract alleviates type 2 diabetes in mice via modulating gut microbiota-host co-metabolism of branched-chain amino acid. Phytother. Res. 37, 3195–3210. Epub 2023 Apr 4. 10.1002/ptr.7822 37013717

[B47] Zhu Y. Alvares K. Huang Q. Rao M. S. Reddy J. K. (1993). Cloning of a new member of the peroxisome proliferator-activated receptor gene family from mouse liver. J. Biol. Chem. 268, 26817–26820. 10.1016/s0021-9258(19)74184-2 8262913

